# Detection of Mouse Cough Based on Sound Monitoring and Respiratory Airflow Waveforms

**DOI:** 10.1371/journal.pone.0059263

**Published:** 2013-03-21

**Authors:** Liyan Chen, Kefang Lai, Joseph Mark Lomask, Bert Jiang, Nanshan Zhong

**Affiliations:** 1 Department of Respiratory Diseases, The 1^st^ Affiliated Hospital of Guangzhou Medical College, Guangzhou, China; 2 Guangzhou Institute of Respiratory Disease, Guangzhou, China; 3 State Key Laboratory of Respiratory Disease, Guangzhou Medical College, Guangzhou, China; 4 Buxco Electronics, Inc, Wilmington, North Carolina, United States of America; Charité Universitaetsmedizin Berlin, Germany

## Abstract

Detection for cough in mice has never yielded clearly audible sounds, so there is still a great deal of debates as to whether mice can cough in response to tussive stimuli. Here we introduce an approach for detection of mouse cough based on sound monitoring and airflow signals. 40 Female BALB/c mice were pretreated with normal saline, codeine, capasazepine or desensitized with capsaicin. Single mouse was put in a plethysmograph, exposed to aerosolized 100 µmol/L capsaicin for 3 min, followed by continuous observation for 3 min. Airflow signals of total 6 min were recorded and analyzed to detect coughs. Simultaneously, mouse cough sounds were sensed by a mini-microphone, monitored manually by an operator. When manual and automatic detection coincided, the cough was positively identified. Sound and sound waveforms were also recorded and filtered for further analysis. Body movements were observed by operator. Manual versus automated counts were compared. Seven types of airflow signals were identified by integrating manual and automated monitoring. Observation of mouse movements and analysis of sound waveforms alone did not produce meaningful data. Mouse cough numbers decreased significantly after all above drugs treatment. The Bland-Altman and consistency analysis between automatic and manual counts was 0.968 and 0.956. The study suggests that the mouse is able to present with cough, which could be detected by sound monitoring and respiratory airflow waveform changes.

## Introduction

Cough is one major defensive reflex that enables vital clearance of secretions and harmful elements from the respiratory tract. Each involuntary cough event involves a series of activities in an integrated reflex arc. Capsaicin has been shown to act mainly on capsaicin-sensitive fibers. A number of authors have proposed that neurogenic inflammatory mediators from endings of capsaicin-irritated C fibers act on rapid adaption receptors, which in turn generate the stimuli that ultimately give rise to cough [1]–[3].

Despite their widespread use in mechanistic studies or in new drug trials for cough [4]–[5], guinea pig cough models have such experimental limitations as high costs, physical weakness and a large demand for experimental drugs. Especially, a guinea pig has 32 couples of chromosomes, which is different from the 21 couples in a human being. In this regard, mice can be more suitable owing to shorter reproductive cycle, prolificacy, less demand for feeding and drugs, and readiness for genetic manipulations. Furthermore, mice have 20 couples of chromosomes, which share 80% of hereditary substances and 99% of genes with human beings. Therefore, detection of cough in mice seems promising.

But there is still controversy over whether mice can cough in response to tussive stimuli [6], chiefly because of their tiny anatomic structures and weak sound signals reported in very few studies with mixed results. In studies from India and Mexico, mice were exposed to irritants and then placed in an up-ended filter funnel with a stethoscope at the tip to be monitored for cough sounds. Since the sounds were not recorded for further analysis, conclusions of these studies appeared somewhat arbitrary [7]–[8]. In experiments by Junzo Kamei who has persisted in anti-tussive studies in mice models for more than two decades, a double-chamber plethysmograph was employed in which the head and body of a consciously restrained mouse were positioned respectively in each of the two separated chambers. The cough in mice was determined by altered breaths as measured by pneumotachography in combination with rapid abdominal twitches [9]–[11]. But these studies are not convincing enough because they lacked sound monitoring. Because detection of cough in mice has never yielded clearly audible sounds as found in guinea pigs, it is not generally recognized.

After intensive studies on neurophysiology over the recent years, vagal sensory neurons have been found to exist in mice, and vagus nerve stimulation or vagotomy has been shown to increase or reduce levels of neuropeptides [12]–[14]. Zhang et al recorded single unit activities in the cervical vagus nerve stimulated with bipolar electrodes in mice, and demonstrated the presence of mechanosensors as well as of chemosensors [15]. Transient receptor potential V1 (TRPV1), the main receptor of capsaicin that was found to mediate capsaicin-induced cough [16], was also localized in mice, as indicated by Symanowicz et al [17]who studied respiratory reflex induced by several inhaled irritants in TRPV1 gene knock-out mice. Based on these findings, it can be conclusive that mice possess a similar set of airway sensors and pulmonary reflexes as typically found in larger animals. Therefore, we designed an approach to detecting cough based on sound monitoring and observation of airflow signals in freely moving mice.

## Methods

### Animals

Ten-week-old female BALB/c mice (22–25 g, Guangdong Laboratory Animal Center) were housed in a specific pathogen-free animal facility at the Guangzhou State Key Laboratory of Respiratory Diseases. The animals had free access to food and water, and were maintained at a 12-h light/12-h dark cycle. This study was carried out in strict accordance with the recommendations in the Guide for the Care and Use of Laboratory Animals of the State Key Laboratory of Respiratory Disease. All experimental procedures were approved by the Animal Ethics Committee, The First Affiliated Hospital, Guangzhou Medical College (Approval ID: 00021376).

### Equipments

**Figure 1:**
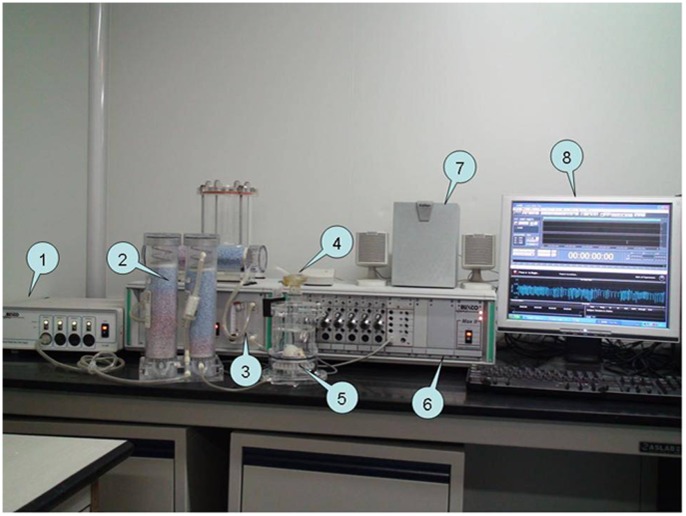
Mouse cough detection equipments. (1) Bias flow generator; (2) Desiccant; (3) Nebuliser controller; (4) Nebuliser; (5) Plethysmograph; (6) Amplifier; (7) Speakers; (8) Monitor display.

For each measurement, a free-moving mouse was put alone within a whole body plethysmography chamber (Buxco Electronics, Inc. Wilmington, NC, USA). A set of software for automated detection and counting of cough events (Finepointe™, jointly developed by Guangzhou State Key Laboratory of Respiratory Diseases and Buxco Electronics, Inc.) was employed to analyze the waveforms of pressure generated from activities of the mouse (such as breaths, coughs, and body behavior) within the chamber. The fluctuations in pressure were reflected on a pneumotachography as the airflow running into and out of the plethysmograph. As such, waveforms inside the chamber can be recorded for real-time analysis and later review (Fig. 1). Moreover, a mini-microphone was mounted to the lateral aperture of plethysmograph to facilitate real-time acoustic monitoring. Sound and sound waveforms were recorded by the software Adobe Audition (formerly Cooledit Pro, Adobe Systems, California, USA) for further analysis.

### Detection of Cough

The mouse was rendered conscious and free to move in the chamber. The irritant for eliciting cough was prepared by dissolving capsaicin in a solution containing 10% ethanol and 10% Tween-80, tittered at a final concentration of 100 µmol/L. Then we exposed the mouse to aerosol of 1 ml capsaicin from a nozzle for 3 min. The number of cough events elicited in mice was counted during the 3 minutes of and within 3 minutes after nebulized capsaicin stimulation. Briefly, an operator was designated to identify the cough sounds by ear and to observe mouse body movements, in parallel with automated recognition of cough events by Finepointe software during the same procedure. Throughout the detection of cough in mice, raw acoustic signals and box flows were acquired simultaneously and displayed on a computer screen. Abnormal box flows identified as arising from coughs were automatically displayed in white. When a cough sound was clearly heard, the operator immediately hit a hot key which in turn prompted red hollow dots and the word ‘cough’ on the respiratory channel to mark a manually counted cough event. Denoised and amplified sound waveforms through Adobe Audition software were also analyzed to identify the correction of manual cough counts. The operator did not know the automatic monitoring results during the observation period. After the 6 min acoustic monitoring, automatic and manual cough counts were evaluated. A single cough was confirmed only with consistent labeling by both approaches (video S1).

### Antitussive Assay

For antitussive pretreatment, 30 mice were randomly divided into three groups: normal saline (NS) control group, codeine (CDI, Qinghai Pharmaceutical Co. Ltd., China) group and capsazepine (CPZ, a competitive antagonist of capsaicin, Sigma Chemical Co., USA) group (n = 10 in each group). On days −2, −1 and 0, the mice received once-daily antitussive pretreatment with gavage of 0.2 ml normal saline (NS group) or codeine (100 mg/kg, CDI group), or intraperitoneal injection of 0.2 ml capsazepine (6 mg/kg, CPZ group, Sigma Chemical Co., USA). Cough detection was performed at 1 h after the last pretreatment.

### Desensitization of C-fibers by Capsaicin Pretreatment

A group of mice (n = 10) was assigned to receive subcutaneous injection of capsaicin (CAP) at a total dose of 300 mg/kg, scheduled as 50 mg/kg on day −3, 100 mg/kg on day −2, 150 mg/kg on day −1. Pentobarbital sodium (60 mg/kg, i.p.), terbutaline (0.1 mg/kg, s.c. AstraZeneca Pharmaceutical Co. Ltd, UK) and aminophylline (25 mg/kg, i.p. Baiyunshan Pharmaceutical Co. Ltd., China) were given to counteract potential adverse effects associated with the capsaicin injections. Cough detection was performed at 24 h after the drug pretreatment.

### Statistical Analysis

The means of multiple samples were examined with one-way analysis of variance and homogeneity of variance test, followed by least significant difference test, Tamhane post hoc test, or independent samples T-test. Statistical analysis was performed with SPSS software version 12.0 (SPSS Inc., Chicago, IL, USA). Bland-Altman analysis and consistency analysis were performed between automatic and manual cough counts.

## Results

### Airflow Signals of Respiration in Mice

**Figure 2:**
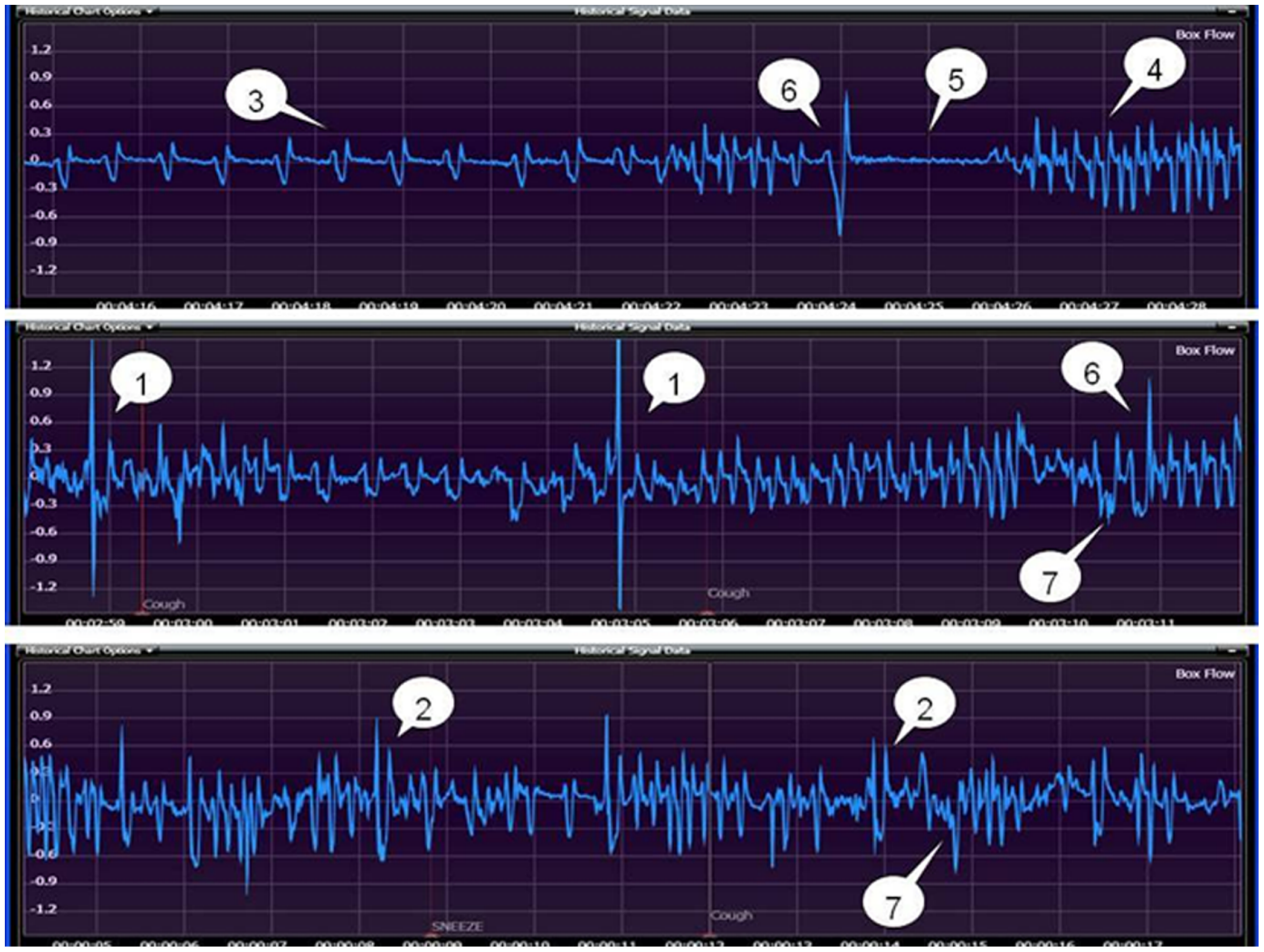
Seven types of mice respiratory waveforms: (1) cough; (2) sneeze; (3) eupnea; (4) tachypnea; (5) breath-holding; (6) deep inspiration; (7) head-twitch.

By holistic evaluation of the acoustic monitoring and Finepointe-based automated detection, seven types of airflow signals of respiration in mice after capsaicin stimulation were defined and identified as follows (Fig. 2):

1. Cough, characterized by apparently enormous amplitudes (pressure changes) and widths (time phase) associated with abrupt head-tossing, opened mouth, abdominal jerking and with a clearly audible sound in mice.
2. Sneeze, characterized sometimes by acoustic waveforms and body behavior (head-tossing, opened mouth, abdominal jerking) similar to those in cough, but chiefly by significantly lowered magnitude of the airflow signals, as well as by dull or little sound.
3. Eupnea, characterized by uniform frequency and depth of airflow signals, as was often seen when mice had accommodated themselves to the chamber environment or recovered from the capsaicin stimulation.
4. Tachypnea, characterized by higher frequency and magnitude of respiration signals, as was often seen when mice were new to the chamber and not familiar with the internal environment, or in rapid movements, or in the process of recovery phase after capsaicin stimulation.
5. Breath-holding, characterized by a significant reduction in both frequency and magnitude of respiration signals to nearly the baseline, as was often seen when the mice voluntarily held back their breath to avert capsaicin inhalation.
6. Deep inspiration, characterized by airflow signals that appeared wider during the early phase and became narrowed later, in contrast to those produced by coughs. In some cases, the signals showed great amplitudes associated with cough-like body behavior (head-tossing, opened mouth and abdominal jerking) but were not accompanied by a cough sound.
7. Head-twitch, accompanied by cough-like sound but also by production of inverted V-shaped airflow signals that were readily distinguishable from those of cough.

### Antitussives Effects

Adverse effects (such as depression and loss of appetite) were shown among the 8 mice that survived out of 10 in the capsaicin group but not in the other groups. Mice subjected to antitussive pretreatment had significantly less cough in response to capsaicin stimulation, compared with the control group (Table 1).

**Table 1:** Frequency of mice cough after antitussive pretreatment compared with control group.

Groups	Cough numbers (/6 min)	P (compared with control group)
Control (n = 10)	17±5	
Codeine (n = 10)	7±4**	0.000
Capsazepine (n = 10)	9±6**	0.005
Capsaicin (n = 8)	4±4**	0.000

Data were expressed as mean ± standard error of the mean (SEM). *P*<0.05 was considered as the level of statistical significance.

** p<0.01, antitussive pretreatment groups had statistical significance compared with the control group.

### Comparison of Automated and Manual Cough Counts

**Figure 3:**
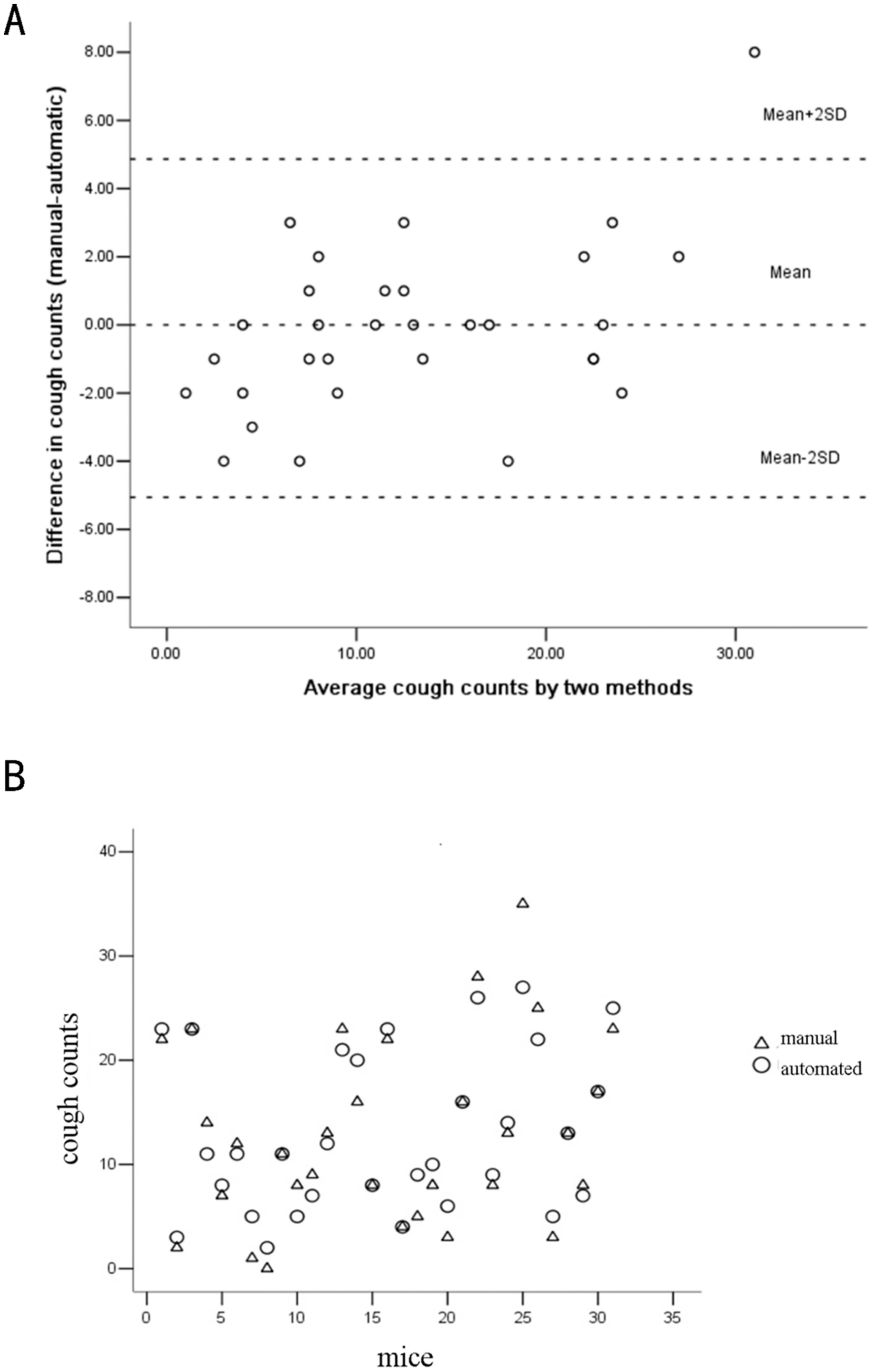
Comparison of automated and manual mouse cough counts. (**A**) Bland-Altman analysis. (B) Consistency analysis. Round circle represents automated cough counts; triangle indicates manual cough counts. The intra-class correlation coefficient (ICC) was 0.956 (95% confidence interval: 0.911∼0.978).

Complete data for 31 out of 38 mice were available for statistical processing, because computer malfunction had led to a loss of the stored data for 7 mice. Bland-Altman analysis showed mean+2SD = 4.87 and mean–2SD = −5.06. Only one pair of the total 31 manual and automatic cough counts (3.23%) had the deviation with 8, and the other 30 pairs were in the limits of agreement (Fig. 3A). Consistency analysis showed that the intra-class correlation coefficient (ICC) was 0.956 (95% confidence interval: 0.911∼0.978). Errors of other sources accounted for 4.4% of the total errors (1-ICC) (Fig. 3B). Both measurements indicated high precision of and good consistency between automated and manual cough counts.

### Criteria for Detection of Cough in Mice

Based on real-time acoustic monitoring and airflow signals in this study, we proposed an established cough in mice should fulfill the following:

1. automatic capture of cough airflow signals, and
2. a clear cough sound audible to the operator and consistent with the captured cough airflow signals.

## Discussion

In the present study, when the airway of a mouse was exposed to a respiratory irritant such as nebulized capsaicin, it responded with a violent reflex to clear the airway and expel the irritant. This reflex can be observed by monitoring the airflow signals, called the box flow. The box flow signal measures the flow of air displaced by the expansion and contraction of air moving in or out of the lungs of the mouse within the plethysmograph. In the lungs, there are two effects which cause the air to expand or contract: heat and humidity added or removed from the air, and air compression or rarefaction. When air is drawn into the lungs, it is heated and humidified. The reverse is true when the air exits the lungs. Heating and humidifying causes the air to expand according to the Combined Gas Law, forcing air out of the plethysmograph. In order to draw air into the lungs, the diaphragm contracts, causing a negative pressure in the lungs. This rarefaction of air in the lungs is the force which moves the air into the lungs. However, the volume of air which is rarefied expands. During expiration, the diaphragm relaxes which causes the air in the lungs to compress in order to force it out. This compression causes the air in the lungs to contract. When the air in the lungs is contracted, air is drawn into the plethysmograph.

During a eupnea, the mouse will draw the air into the lungs with relative ease, without developing much pressure in the lungs. So, the box flow signals during a eupnea are dominated by the temperature and humidity effects. When air is drawn into the lungs, the air will expand and force the rest air out of the chamber. So, inspired flow is observed during a eupnea when the box flow signal trace is below zero.

Typically, a cough is defined as having 3 phases: an inhalation phase, a compression phase, and a forced exhalation phase. The inhalation phase is a deep inspiration. The compression phase may be very short, and occurs when an animal forces an exhalation against the closed glottis. Air pressure is built up which compresses the air in the lungs, drawing air into the chamber, rapidly shown as a high spike on the box flow signal. The mouse then releases the glottis, and continues the expiration. In a normal expiration, air is drawn into the chamber, but during a cough, the air which was compressed during the compression phase, is released during the exhalation phase, and so expands, forcing air out of the chamber. This expansion occurs extremely fast, and can be observed as an enormous negative-going spike on the slope of the box flow signals (indicated in the Decompress & Expire region) [18].

A sneeze is meant to clear the nasal cavity of irritants. The sneeze could be similar to the cough, except that its compression phase is not so clearly defined. Air is compressed, but not by the glottis to completely close off the airway. Instead, the soft palate, uvula, and tongue work together to partially close off the mouth, and create resistance. In addition, the exhaled air is exhaled through both the nose and the mouth. The opening of the glottis in mice can be marked by the steep transient pressure inside the chamber (box pressure) which is indicative of cough. Events with high compression of air also tend to indicate a cough, while sneezing may produce relatively lower compression as pressure is built against nasal resistance only.

**Figure 4:**
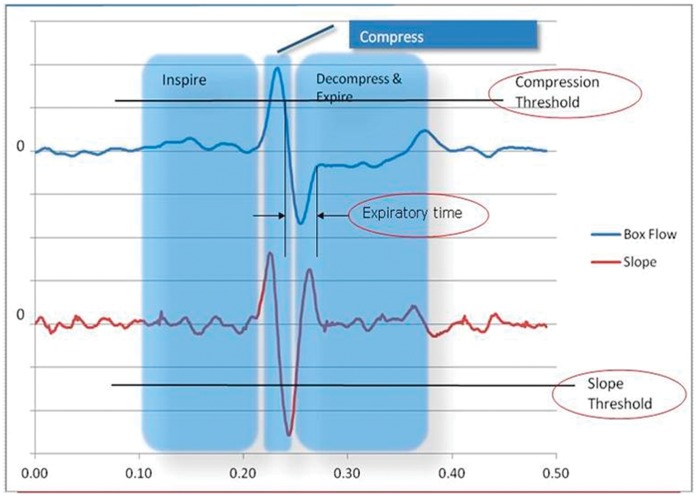
Automated analysis of mouse cough waveforms. The superior blue part indicates mice cough respiratory waveform and the inferior part shows airflow rate slope replot. Coughs were judged based onthe compression threshold, slope threshold and expiratory timephase.

Figure 4 shows the cough signals measured while a mouse was in the chamber. The blow trace is the box flow signal waveforms, and the retrace is the slope of the box flow. On the box flow signals trace, air is forced out of the chamber when the trace is below zero. Above zero, air is drawn into the chamber. Three parameters – compression threshold, slope threshold and exhalation duration, were used for analysis of cough in mice. The thresholds were re-evaluated continuously based on the data collected. Each time the slope threshold was evaluated, the magnitude was rendered no less than a certain value (indicated in the algorithm settings xml as Negative Threshold = “−50”) empirically set to ensure that an analyzer would not respond to noise when the chamber was empty. The magnitude of this value is significantly larger than a slope that occurs during a typical breath, to minimize any type of error. Since the thresholds can be self-adjusting, the algorithm will adjust itself appropriately for different animal weights. The combination of these 3 parameters discriminates between coughs and sneezes based on principles of fuzzy logic. In spite of some differences in methodology, the signal waveforms of cough are similar to those in guinea pigs [18].

During a deep-inspiration, the mouse may take in a very deep breath, and release it without much force. A deep-inspiration will not much increase the box flow signal magnitude, and like a eupnea, will not develop much pressure in the lungs. So the deep-inspiration is also dominated by the temperature and humidity effects. Similarly to the eupnea, inspired flow occurs when the box flow signal trace is below zero.

**Figure 5:**
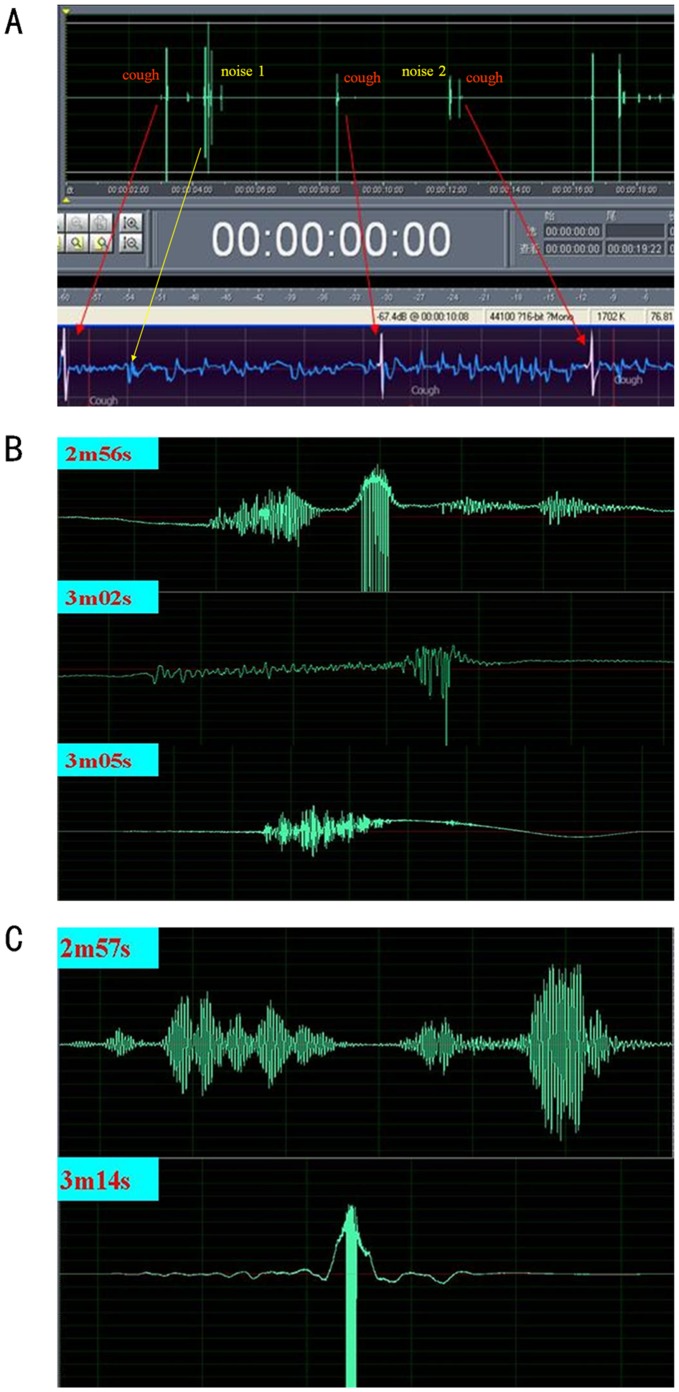
Recorded and amplified sound waveforms according to mouse cough and noises. (A) The upper panel shows sound waveforms in green, and the bottom one shows air flow signals in blue. Cough signals defined by Finepointe software were displayed white color. Manual defined coughs were marked with “cough” and red hollow dots on the screen. Coughs were pointed with red arrows. According to the cough signals, cough sound waveforms were certified. Noise 1 pointed with yellow arrow was head-twitch sound. Noise 2 was the sound of knocking at chamber wall with no abnormal respiratory signals. (B) Three different types of amplified cough sound waveforms distinguished by Cooledit software. (C) Two types of noise sound waveforms, which are different in shape, but similar to cough ones.

When moving freely inside the plethysmograph, the mice may make sounds from frequent maneuvers such as nose-scratching, teeth-tapping, sniffing, and knocking at the walls of chamber. When raw acoustic waveforms appear sound-intensive, or noisy, a cough may be difficult to identify. We then used the Adobe Audition software for a couple of denoising and amplification of waveforms (Fig. 5*A*). In this way, amplified waveforms of defined coughs were scrutinized and compared with those of noises. We found a wide range of variations in the waveforms of both coughs (Fig. 5*B*) and noises (Fig. 5*C*), which were diverse and not easily discriminated from each other by appearance. This means a difficulty in accurately identifying coughs from a free-moving mouse simply by acoustic waveform analysis. On the other hand, these noises were apparently different and therefore well distinguished from coughs through a microphone to the ears of an operator. Moreover, the noises did not appear to be accompanied by abnormal box flows (Fig. 5*A* noise 2), and were thus well differentiated real-time from coughs by respiratory waveforms (audio S1).

**Figure 6:**
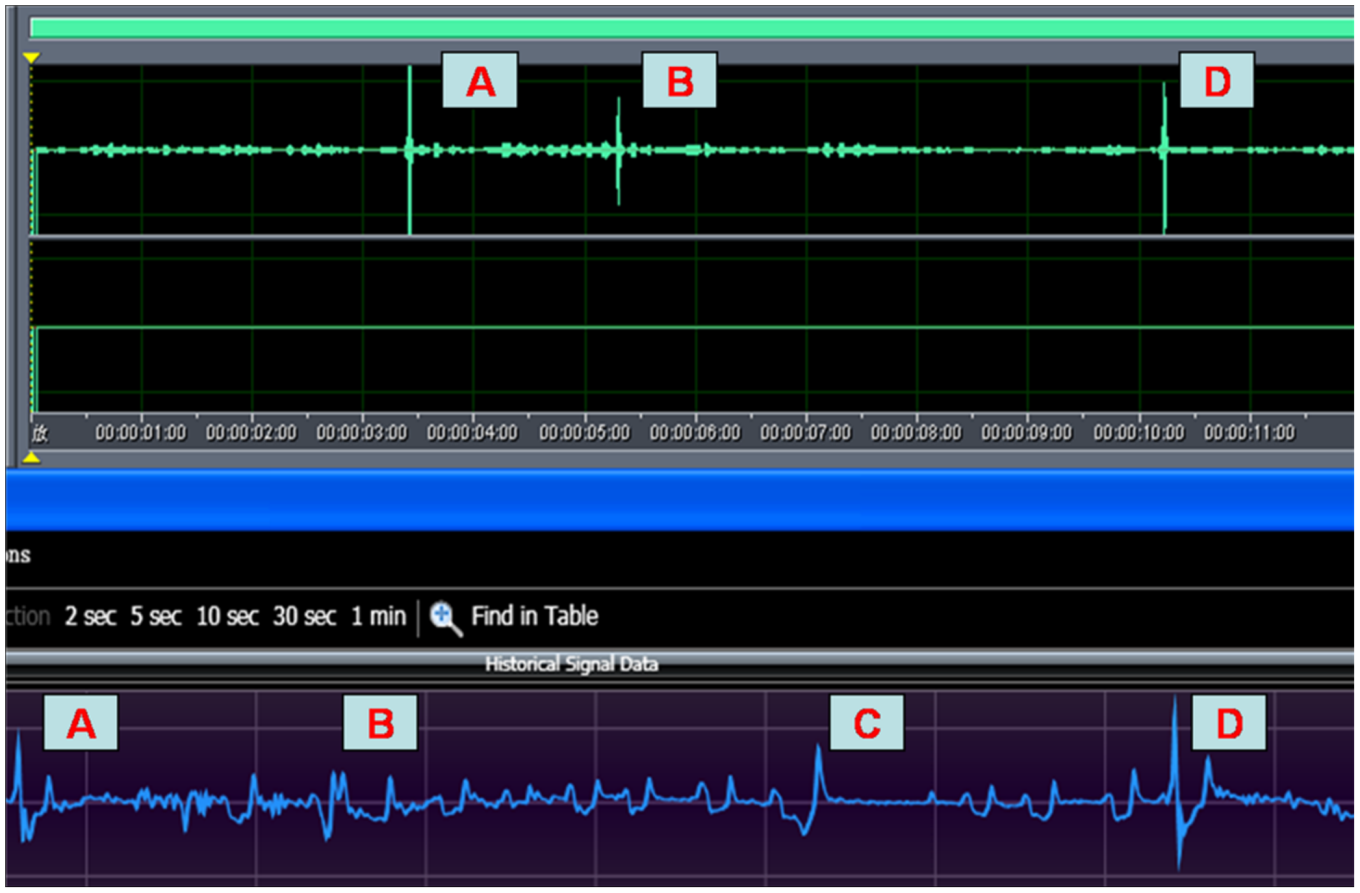
Deep inspiration could be distinguished from coughs through the shape of respiratory signals. Deep inspiration with air flow signals that appeared wider during the early phase and became narrowed later, in contrast to those produced by coughs. Head-twitch, accompanied with a cough-like sound but producing inverted V-shaped air flow signals that was readily distinguishable from coughs. A: Cough; B: head-twitch; C: deep inspiration; D: cough.

Body behavior of mice was also observed carefully throughout the experiment. We found noted head-tossing, opening mouth, or rapid jerking of abdominal muscles among the mice when coughing was also present in deep inspiration alone. This indicates that cough in mice cannot be judged simply through body behavior. Deep inspiration may lead to high-amplitude box flow signals which sometimes can be automatically mislabeled as coughs, but the signals are substantially different in shape from those produced by cough (Fig. 6, audio S2), and not associated with cough sounds. Although head-twitching in mice may generate loud, cough-like sounds, the side-to-side movements of head-twitching differ from the down-and-up head-tossing which frequently accompanies the cough. Moreover, the V-shaped waveforms during head-twitching can be easily distinguished from a cough response on the respiratory channel (Fig. 5*A* noise 1 and Fig. 6). An operator could help in discrimination of these events when necessary, suggesting the importance of manual monitoring.

As one of narcotic anti-tussives and the currently accepted gold standard for cough suppressant therapy, codeine is believed to suppress the responsiveness of one or more components of the central reflex pathway for cough by activating the *µ*- or k-opioid *receptors in brain tissues*. Moskowitz and Goodman successfully used a quantitative in vitro autoradiography procedure in demonstrating the presence of *µ*-opioid receptors in the central nervous system of mice that mediate the anti-tussive actions of codeine [19]. Capsaicin, the pungent component found in hot chili peppers, has been shown to have a unique excitatory action on a sub-population of afferent sensory neurons [20]–[22]. Pretreatment with a large-dose of capsaicin could deplete neuropeptides of the vagus, resulting in lowered neurosensitivity and hence less cough in response to a second capsaicin challenge. Bevan and coworkers found that the effects of capsaicin might also be neutralized by capsazepine, a synthetic capsaicin analogue and a competitive antagonist of TRPV1 receptors [23]. In our study, pretreatments with codeine, capsazepine and large-dose capsaicin were all shown to significantly reduce coughs in mice exposed to capsaicin stimulation, compared with the normal saline group.

Totally, software-based automated detection revealed box flows accounting for cough. The cough of mice sounded clear and brassy, readily recognized by human ears. On the contrary, the greatly variable acoustic waveforms or confusing body behavior appeared less valuable for analysis or detection of cough in mice. On the basis of sound monitoring, we worked out the criteria for identification of cough in mice, which included: 1) automated capture of cough air flow waveforms (automatic monitoring), and 2) a clear cough sound audible to an operator and consistent with the captured cough air flow waveforms (manual monitoring). Manual monitoring may make a difference in the detection in some cases, when box flows of atypical cough might show low amplitudes and were therefore not labeled as cough automaticlly, they were still determined to be of actual coughs by the operator owing to clearly audible cough sounds. To date, combination of manual monitoring and measurement of box flows may be the optimal method for cough detection in mice.

The present study has some advantages in cough detection in mice over the previous reports. Firstly, use of free-moving mice can render settings of this study closer to a natural condition. Secondly, cough sounds in mice were monitored directly by human ears and recorded for later analysis, which may provide a keystone for detection of cough in mice. Thirdly, precise classification of box flows into 7 types may facilitate better identification of cough. Fourthly, since we attached less importance to body behavior of mice in identifying a cough, subjective errors by an operator could be minimized. Fifthly, as this study has indicated, there may be no necessity to analyze acoustic waveforms. In our previous study, a 6-min record would require 4 hours or so for later analysis, a labor-intensive and time-consuming task. Now, an operator can listen real-time for any possible cough sound and confirm it with reference to the box flow. This will allow for much less workload, quicker processing, and acquisition of more accurate data. Furthermore, the method of mouse cough detection established in this study is closer to normal physiology, less expensive, and easy to reproduce. In general, our new methodology of detecting mouse cough seems promising for widespread use in the future research.

Consistency between automated and manual counts reached over 95% in the present study. The good agreement also suggests that multiple mice could be assessed simultaneously so that the time needed is reduced and batches of experimental animals could be executed easily.

In conclusion, cough in mice as a response to capsaicin was successfully elicited in the present study. The establishment of mouse cough detection method based on manual acoustic monitoring and automated monitoring of box flows can not only confirm the concept that “the mouse has cough”, but also may be useful for mechanistic study, new anti-tussives development and optimization of cough-related animal models.

## Supporting Information

Video S1 **Real time mouse cough detection process.**
(MP4)

Audio S1 **According to the cough respiratory waveforms, we found the amplified sound waveforms.** Noise 1 was the sound of head-twtich, the sound was similar to cough. But body movements were shaking head from left to right, which were different from raising head while coughing. Especially, the respiratory waveforms displayed “V” shape which was different from cough ones. Noise 2 was the sound of knocking chamber wall. When moving freely inside the plethysmograph, the mice may make sounds from frequent maneuvers such as nose-scratching, teeth-tapping, sniffing, et al. These sounds and body movements were so different from cough that could be distinguished by manipulator at real time. Otherwise, the noises produced different or no respiratory waveforms, and were thus well differentiated real-time from coughs using the Finepointe software.
(MP3)

Audio S2 **Here C is the deep inspiration waveform.** The body movement was similar, but the respiatory waveforms were different from coughs and with no sound. This hints: mice cough could not accuratedly judged through mice movement observation.
(MP3)
